# The Impact of Hypertension Definition Based on Two-visit Strategy on Estimate of Hypertension Burden: Results From the China Health and Nutrition Survey 1989–2011

**DOI:** 10.2188/jea.JE20190163

**Published:** 2021-03-05

**Authors:** Ying-Li Liu, Ying-Jun Mi, Bing Zhang, Hui-Jun Wang, Jie Yu, Xing-Bing Pan, Chao Wang, Qing-Bao Tian

**Affiliations:** 1 Department of Epidemiology and Statistics, School of Public Health, Hebei Medical University, Shijiazhuang, China; 2 Hebei Province Key Laboratory of Environment and Human Health, Shijiazhuang, China; 3 Department of Social Medicine and Health Care Management, School of Public Health, Hebei Medical University, Shijiazhuang, China; 4 National Institute for Nutrition and Health, Chinese Center for Disease Control and Prevention, Beijing, China

**Keywords:** hypertension definition, two-visit strategy, hypertension burden, Chinese adults

## Abstract

**Background:**

The diagnosis of hypertension should be based on the mean of two or more properly measured BP readings on each of two visits for clinical practice, but a one-visit strategy was applied in most epidemiological surveys. The impact of hypertension definition based on two visits on estimates of hypertension burden is unknown. This study aims to assess the impact of hypertension diagnosis based on a two-visit strategy for estimating hypertension burden in China.

**Methods:**

The one-visit and two-visit strategies were applied to investigate the incidence of hypertension in a cohort study based on the China Health and Nutrition Survey (CHNS) 1989–2011. Additionally the prevalence of hypertension was investigated in a cross-sectional study based on the CHNS 2006–2009/2011 and the hypertension burden in China was estimated with data from the 2012–2015 China hypertension survey.

**Results:**

Overall, the age-adjusted incidence of hypertension based on the two-visit strategy (1.82%; 95% confidence interval [CI], 1.74–1.90%) was 62.1% lower than estimation based on the one-visit strategy (4.80%; 95% CI, 4.68–4.93%). Similar results were found in the prevalence of hypertension (one-visit: 18.13% [95% CI, 17.34–18.92%]; two-visit: 9.47% [95% CI, 8.87–10.07%]). When the two-visit strategy was applied to the 2012–2015 China hypertension survey, the hypertension burden was predicted to be overestimated by 25.5–47.8% (based on JNC 7) and 23.5–48.2% (based on the 2017 ACC/AHA).

**Conclusion:**

The hypertension burden would decrease from 244.5 million persons to 127.5–182.3 million persons in China if the two-visit strategy was applied.

## INTRODUCTION

Measured blood pressure is intrinsically variable because every cardiac cycle produces a different blood pressure, and it is characterized by considerable variation within and between days.^1^^–^^3^ The diagnosis of hypertension should be based on the mean of two or more properly measured BP readings on each of two or more visits for clinical practice.^4^^,^^5^ However, implementation of the recommendation diagnosis in large epidemiological surveys is very difficult. In most of the epidemiological surveys, the one-visit strategy (1–3 measurements) was applied to estimate hypertension burden. In China, the burden of hypertension is increasing along with rising incomes, urbanization, and aging of the population in recent decades.^6^ Data from the China Hypertension Survey (2012–2015) showed that 23.2% of Chinese adults (estimated at 244.5 million persons) were hypertensive, and the prevalence of pre-hypertension was 41.3% (estimated 435.3 million) based on one-visit strategy.^7^

The diagnostic criteria of hypertension based on the one-visit strategy might cause subjects with high blood pressure (BP) variability regression to the mean,^8^ white-coat hypertension, and episodic hypertension, leading to a final overestimation of hypertension burden. Some studies reported that, based on the one-visit strategy, the prevalence could be overestimated by 6–12.6%.^9^^,^^10^ Nowadays in China, the one-visit strategy is still applied in most of the surveys of hypertension, and the impact on hypertension burden is unknown.

This study aims to investigate the impact of hypertension definition based on the two-visit strategy of estimating hypertension burden. On the basis of data obtained from China Health and Nutrition Survey (CHNS) 1989–2011, both a cohort study and a cross-sectional study were conducted to investigate the discrepancy between the one-visit strategy and the two-visit strategy.

## METHODS

### Study design

The CHNS was a large-scale, longitudinal, household-based survey in China.^11^^,^^12^ It was conducted in 1989, 1991, 1993, 1997, 2000, 2004, 2006, 2009, and 2011. Twelve provinces or municipalities were included in this survey, which accounts for approximately 56% of China’s population, varying substantially in geography, economic development, public resources, and health indicators.^13^ A detailed description of the survey design and procedures has been published elsewhere.^12^

To evaluate the impact of hypertension definition on the estimates of hypertension burden, both a dynamic cohort study and a cross-sectional study were conducted. Based on the data from CHNS 1989–2011, one-visit (1–3 measurements) and two-visit strategies were applied to investigate the incidence of hypertension in a cohort study. In addition, using these two strategies, a cross-sectional study based on CHNS 2006 and CHNS 2006–2009/2011 was conducted to investigate the prevalence of hypertension. Based on the data from the 2012–2015 China hypertension survey,^7^ the hypertension burden in China was estimated using these two strategies (according to the JNC 7 high blood pressure guideline criteria and the 2017 ACC/AHA high blood pressure guideline criteria).

### Population

Analysis was based on data from adults aged ≥18 years, providing information on age, sex, and detailed physical examinations, including weight, height, and blood pressure. In the cohort study, data were obtained from seven waves of the CHNS during 1989–2006 at baseline, and normotensive participants were followed up during 1991–2009 as the first-visit/one-visit strategy. Correspondingly, the new cases of hypertension in the one-visit strategy were still followed up as the second-visit strategy from 1993–2011. Once the new cases were diagnosed with hypertension again, they were considered as the final new cases of the two-visit strategy. In a cross-sectional study, analysis was based on data from CHNS 2006 at baseline as the first-visit/one-visit. Hypertensive patients in 2006 were followed up to 2009 or 2011 as the second visit. Correspondingly, the cases once still diagnosed with hypertension were considered as the hypertensive patients of the two-visit strategy.

To limit biases caused by preexisting factors, analysis excluded participants who were pregnancy or lactating, participants with missing information about blood pressure, participants with a difference in systolic and diastolic pressures of <10 mm Hg or with extreme or implausible height (<120.0 cm) or body mass index (BMI) (<15.0 kg/m^2^ or >40.0 kg/m^2^).

### Measurements and definition of hypertension

Data on age, gender, height, weight, smoking, drinking, region, blood pressure, and use of antihypertensive medication were collected. The body weight of participants dressed in light clothing was measured without shoes to the nearest 0.01 kg with a calibrated beam scale (Seca North America, Chino, CA, USA). The height of barefoot subjects was measured to the nearest 0.1 cm, using a portable stadiometer (Seca North America). Blood pressure (BP) was the mean of three measurements collected after 10 min of seated rest.^14^

According to the seventh Joint National Commission guidelines (JNC 7),^15^ hypertension was defined as systolic blood pressure/diastolic blood pressure (SBP/DBP) ≥140/90 mm Hg or current use of antihypertensive medication (Med). In addition, hypertension was classified into four subtypes: (1) isolated systolic hypertension (ISH), defined as SBP ≥140 mm Hg and DBP <90 mm Hg; (2) isolated diastolic hypertension (IDH), defined as SBP <140 mm Hg and DBP ≥90 mm Hg; (3) systolic and diastolic hypertension (SDH), defined as SBP ≥140 mm Hg and DBP ≥90 mm Hg; and (4) current use of antihypertensive medication. According to the 2017 ACC/AHA high blood pressure guideline criteria, hypertension was defined as SBP/DBP ≥130/80 mm Hg or current use of antihypertensive medication.

### Statistical methods

The sample sizes were sufficient to detect an annual increase of 0.05 percentage points with >90% power. Based on the data at the interview, age was grouped into 18–39, 40–59, and ≥60 years; BMI was stratified into <18.5, 18.5–23.9, 24–27.9, and ≥28 kg/m^2^ groups; degrees of elevation of blood pressure were grouped into 140–150/90–95, 150–160/95–100, and ≥160/100 mm Hg.

A time-dependent, Cox proportional hazard regression model with follow-up duration as the timescale was applied, to calculate the hazard ratios (HRs) and 95% confidence intervals (CIs) of the one-visit strategy based on the two-visit strategy.

Taking into account unequal probabilities of selection, both the incidence and prevalence of hypertension were adjusted via the direct method according to the 2010 census of the Chinese adult population, using the corresponding age groups. All statistical analyses were performed using SPSS software for Windows, version 21.0 (IBM Corp., Armonk, NY, USA). The statistical significance was set at a two-tailed *P* < 0.05.

## RESULTS

eTable 1 shows the baseline characteristics of the study population in the cohort study. A total of 14,057 participants aged ≥18 years at baseline were included in the cohort study containing 6,395 males and 7,662 females. The selection procedure of the participants in this study was summarized in eFigure 1. The median age of the participants was 34.8 (interquartile range [IQR], 20.1) years old among the males and 33.4 (IQR, 19.4) years old among the females. The corresponding median blood pressure (SBP/DBP) was 113.7/75.0 mm Hg and 110.0/70.0 mm Hg, respectively.

eTable 2 shows the baseline characteristics of the study population in the cohort study. In the cross-sectional study, there were 9,127 participants (4,304 males and 4,823 females) aged ≥18 years at baseline were included in 2006, and 2,135 hypertensive patients were diagnosed. The selection procedure of the participants was presented in eFigure 2. The median age of the males and females were 49.4 (IQR, 21.6) and 49.6 (IQR, 21.4) years old; correspondingly, the median blood pressure (SBP/DBP) was 120.0/80.0 mm Hg and 119.3/78.0 mm Hg (eTable 2).

### Age-adjusted incidence of hypertension and subtypes in CHNS1989**–**2006 based on one-visit and two-visit strategies in the cohort study according to the JNC 7

Table 1 shows the incidence of hypertension and subtypes in CHNS1989–2006 based on one-visit and two-visit strategies in the cohort study according to the JNC 7. During the mean follow-up time of 5.85 person-years for the first visit as the one-visit strategy, 3,945 new cases of hypertension occurred. The incidence of hypertension for the one-visit was 4.80 (95% CI, 4.68–4.93) per 100 person-years. In addition, the 3,945 new cases of hypertension were continued to be followed up for the second visit as the two-visit strategy, and 1,437 new cases of hypertension still were diagnosed as hypertension. The incidence of hypertension for the two-visit was 1.82 (95% CI, 1.74–1.90) per 100 person-years (the interval between two blood pressure measurements of the two-visit strategy was 3.17 person-years). To elaborate the impact of hypertension definition on estimating hypertension burden, the discrepancy between the incidence of the one-visit and the two-visit strategies was calculated. It revealed that 62.1% hypertension patients, based on the one-visit strategy, could be averted when the two-visit strategy was applied, meaning the effect of the one-visit strategy on overestimating the hypertension incidence.

**Table 1.** Incidence^a^ of hypertension based on one-visit or two-visit strategy during 1991–2009

Characteristics	One-visit strategy	Two-visit strategy	Δ (%)
All participants	4.80 (4.68–4.93)	1.82 (1.74–1.90)	−62.1
Gender			
Male	5.34 (5.15–5.53)	2.06 (1.94–2.18)	−61.4
Female	4.38 (4.21–4.54)	1.64 (1.54–1.74)	−62.6
Age, years			
18–39	2.24 (2.13–2.34)	0.69 (0.63–0.74)	−69.2
40–59	5.41 (5.15–5.67)	2.25 (2.07–2.42)	−58.4
≥60	10.48 (9.72–11.24)	4.00 (3.52–4.49)	−61.8
BMI, kg/m^2^			
<18.5	3.23 (2.90–3.55)	1.03 (0.84–1.22)	−68.1
18.5–23.9	4.52 (4.38–4.66)	1.72 (1.63–1.81)	−61.9
24.0–27.9	6.83 (6.43–7.22)	2.72 (2.46–2.97)	−60.2
≥28.0	9.92 (8.67–11.18)	4.17 (3.33–5.01)	−58.0
Smoking status			
Never smoking	4.59 (4.44–4.73)	1.73 (1.64–1.82)	−62.3
Ever smoking	5.32 (5.08–5.56)	2.03 (1.88–2.18)	−61.9
Drinking status			
Never drinking	4.48 (4.33–4.63)	1.63 (1.54–1.72)	−63.6
Ever drinking	5.45 (5.22–5.68)	2.16 (2.02–2.31)	−60.4
Region			
Urban resident	4.87 (4.65–5.10)	1.89 (1.75–2.04)	−61.2
Rural resident	4.77 (4.62–4.92)	1.78 (1.69–1.87)	−62.7

BMI, body mass index.

^a^Age adjusted incidence rate and 95% confidence intervals of hypertension and subtypes by the direct method to the year 2010 Census population.

Δ = (Incidence rate of hypertension based on two-visit strategy − Incidence rate of hypertension on one-visit strategy)/Incidence rate of hypertension based on one-visit strategy

### Age-adjusted prevalence of hypertension and subtypes in CHNS 2006, based on one-visit and two-visit strategies in a cross-sectional study according to the JNC 7

Table 2 shows the prevalence of hypertension and subtypes in CHNS 2006 based on one-visit and two-visit strategies according to the JNC 7. In the cross-sectional study, 2,135 participants were diagnosed with hypertension as the one-visit strategy. The prevalence of hypertension was 18.13% (95% CI, 17.34–18.92%). Further, 2,135 hypertensive participants were continued follow-up until 2009 or 2011 for the second visit, and 1,180 participants still were hypertensive. The prevalence of hypertension for the two-visit strategy was 9.47% (95% CI, 8.87–10.07%). The discrepancy between the prevalence of the one-visit and the two-visit strategies was 47.8%. Considering the influence of lost participants in the second visit, a sensitivity analysis was carried out in the cross-sectional study. When the lost participants were treated as patients with hypertension, the prevalence in the two-visit strategy was 13.50% (95% CI, 12.80–14.20%), and the discrepancy between the prevalence in the one-visit strategy and the two-visit strategy was 25.5% (eTable 3).

**Table 2.** Prevalence^a^ of hypertension based on one-visit strategy or two-visit strategy in 2006

Characteristics	One-visit strategy	Two-visit strategy	Sensitivity analysis^b^	Δ^(Δ1, Δ2)^ (%)
All participants	18.13 (17.34–18.92)	9.47 (8.87–10.07)	13.50 (12.80–14.20)	(−47.8, −25.5)
Gender				
Male	20.48 (19.28–21.69)	10.14 (9.23–11.04)	14.98 (13.91–16.04)	(−50.5, −26.9)
Female	16.06 (15.03–17.10)	8.91 (8.11–9.73)	12.21 (11.28–13.13)	(−44.5, −24.0)
Age, years				
18–39	5.98 (5.07–6.90)	1.41 (0.96–1.87)	3.44 (2.74–4.15)	(−76.4, −42.5)
40–59	21.16 (19.93–22.38)	12.33 (11.34–13.32)	15.76 (14.67–16.85)	(−41.7, −25.5)
≥60	44.70 (42.66–46.74)	25.24 (23.46–27.02)	36.03 (34.06–38.00)	(−43.5, −19.4)
BMI, kg/m^2^				
<18.5	9.46 (7.04–11.88)	4.84 (3.07–6.61)	6.83 (4.74–8.91)	(−48.8, −27.8)
18.5–23.9	13.92 (12.98–14.87)	6.59 (5.92–7.27)	9.79 (8.98–10.60)	(−52.7, −29.7)
24.0–27.9	22.63 (21.02–24.24)	11.64 (10.41–12.88)	16.49 (15.06–17.92)	(−48.6, −27.1)
≥28.0	34.41 (31.10–37.72)	21.75 (18.87–24.63)	29.51 (26.33–32.69)	(−36.8, −14.2)
Smoking status				
Never smoking	17.36 (16.42–18.30)	9.40 (8.68–10.13)	13.19 (12.35–14.03)	(−45.9, −24.0)
Ever smoking	20.02 (18.55–21.49)	9.75 (8.66–10.83)	14.27 (12.99–15.55)	(−51.3, −28.7)
Drinking status				
Never drinking	16.58 (15.66–17.51)	8.74 (8.04–9.45)	12.49 (11.67–13.31)	(−47.3, −24.7)
Ever drinking	21.19 (19.71–22.68)	10.92 (9.79–12.05)	15.34 (14.03–16.65)	(−48.5, −27.6)
Region				
Urban resident	18.74 (17.38–20.11)	9.54 (8.51–10.57)	14.42 (13.19–15.66)	(−49.1, −23.1)
Rural resident	17.78 (16.81–18.74)	9.46 (8.72–10.20)	13.00 (12.15–13.85)	(−46.8, −26.9)

BMI, body mass index.

^a^Age adjusted prevalence and 95% confidence intervals of hypertension using the direct method to the year 2010 Census population.

^b^Prevalence based on two-visit strategy = (the population of hypertension based on two-visit strategy + the lost population based on two-visit strategy)/all the population in 2006

Δ1 = (Prevalence of hypertension based on two-visit strategy − Prevalence of hypertension based on one-visit strategy)/Prevalence of hypertension based on one-visit strategy

Δ2 = (Prevalence of sensitivity analysis − Prevalence of hypertension based on one-visit strategy)/Prevalence of hypertension based on one-visit strategy

### Effects of risk factors on the discrepancy of incidence (or prevalence) between the one-visit and two-visit strategies

**Figure 1.**
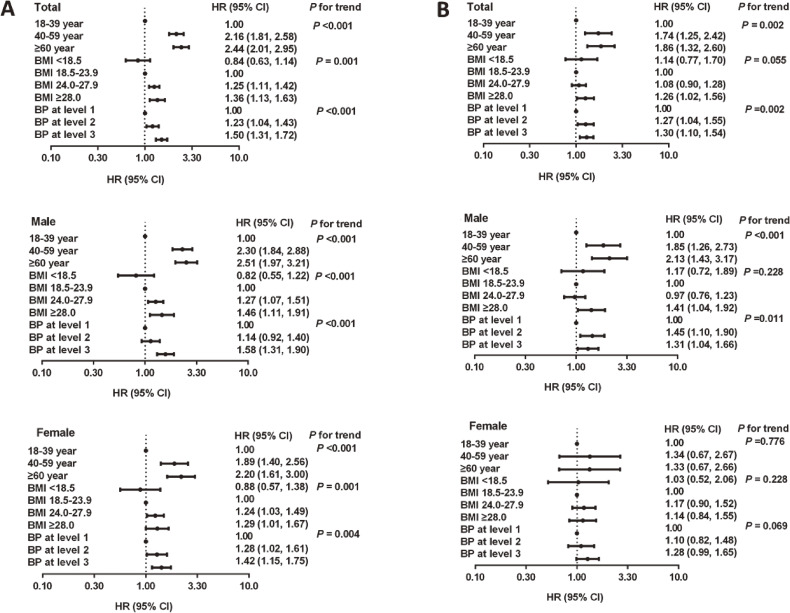
Effects of risk factors on the discrepancy of incidence (or prevalence) between the one-visit and two-visit strategies using Cox’s models: (A) factors associated with the discrepancy of incidence between the one-visit and two-visit strategies; (B) factors associated with the discrepancy of prevalence between the one-visit and two-visit strategies.

To clarify the effects of age, gender, BMI, smoking status, drinking status, region and blood pressure levels further on the discrepancy in the incidence (or prevalence) of hypertension between the one-visit and the two-visit strategies, the adjusted HRs were calculated using the Cox proportional hazards regression model. No significant effect was found in smoking status, drinking status, and region, whereas old age, overweight/obesity, and higher blood pressure were all significantly associated with an increased discrepancy in the incidence of hypertension between the one-visit strategy and the two-visit strategy, especially among males (Figure 1A). Similar trends with weaker effects were observed in the discrepancy in prevalence of hypertension overall and in males (Figure 1B). The effects of old age, overweight/obesity, and higher blood pressure level disappeared in females (Figure 1B).

### The predicted burden of hypertension in China based on JNC 7 high blood pressure guideline criteria and 2017 ACC/AHA high blood pressure guideline criteria

Table 3 shows the predicted burden of hypertension. According to the 2017 ACC/AHA high blood pressure guideline criteria proposed in recent years, the prevalence of hypertension in CHNS 2006 was 38.39% and 19.87%, based on the one-visit and the two-visit strategies, respectively. In addition, when the sensitivity analysis was conducted, the prevalence of hypertension based on the two-visit strategy was 29.38%. As reported that the prevalence of hypertension based on the China hypertension survey (2012–2015) was the latest data in China, the prediction referred to this prevalence. According to the JNC 7 high blood pressure guideline criteria, 244.5 million people were hypertensive in China when the one-visit strategy was applied while the number would decrease to 127.5–182.3 million when the two-visit strategy was adopted. In addition, when the 2017 ACC/AHA high blood pressure guideline criteria was used, there were 489.0 million hypertensive patients based on the one-visit strategy but 254.0–374.1 million when the two-visit strategy was applied.

**Table 3.** Predicted prevalence^a^ based on JNC 7 high blood pressure guideline criteria and 2017ACC/AHA high blood pressure guideline criteria in China

	Prevalence based on CHNS survey 2006			Predicted prevalence based on China hypertension survey (2012–2015)			
	
	Prevalence based on one-visit strategy in CHNS Survey (%)^b^	Prevalence based on two visit strategy^c,d^ (%)	Δ^(^**^Δ1, Δ2^**^)^ (%)	Prevalence in China hypertension survey^e^ (%)	hypertension cases^f^ (million persons)	Predicted prevalence^g^ in China based on two-visit strategy Δ^(^**^Δ1, Δ2^**^)^ (%)	Predicted hypertension cases^h^ based on two-visit strategy (million persons)
**Based on JNC 7 high blood pressure guideline criteria**							
All participants	18.13	(9.47, 13.50)	(−47.8, −25.5)	23.2	244.5	(12.1, 17.3)	(127.5, 182.3)
Male	20.48	(10.14, 14.98)	(−50.5, −26.9)	24.5	130.4	(12.1, 17.9)	(64.4, 95.3)
Female	16.06	(8.91, 12.21)	(−44.5, −24.0)	21.9	114.1	(12.1, 16.6)	(63.1, 87.0)

**Based on 2017ACC/AHA high blood pressure guideline criteria**							
All participants	38.39	(19.87, 29.38)	(−48.2, −23.5)	46.4	489.0	(24.1, 35.5)	(254.0, 374.1)
Male	44.67	(23.45, 34.66)	(−47.5, −22.4)	52.3	278.5	(27.5, 40.6)	(146.4, 216.2)
Female	32.43	(16.61, 24.49)	(−48.8, −24.5)	40.4	210.5	(20.7, 30.5)	(52.5, 103.5)

ACC, American College of Cardiology; AHA, American Heart Association; JNC, Joint National Committee on Prevention, Detection, Evaluation, and Treatment of High Blood Pressure.

^a^Age adjusted prevalence of hypertension by the direct method to the year 2010 Census population.

^b^The prevalence based on one-visit strategy = (the population of hypertension based on one-visit strategy/all the population in 2006)

^c^The prevalence based on two-visit strategy = (the population of hypertension based on two-visit strategy/all the population in 2006)

^d^The prevalence based on two-visit strategy = (the population of hypertension based on two-visit strategy + the lost population based on two-visit strategy)/all the population in 2006

Δ^1^: (a − c)/c

Δ^2^: (b − c)/c

^e^The prevalence reported by Wang et al.^7^

^f^Predicted hypertension cases was calculated based on the year 2010 Census population, e and the prevalence reported by Wang et al.^7^

^g^Predicted prevalence was calculated based on e and Δ^(^**^Δ1, Δ2^**^)^.

^h^Predicted hypertension cases based on f and g.

## DISCUSSION

This study evaluated the effect of the one-visit and two-visit strategies on estimating the incidence as well as prevalence of hypertension among Chinese adults aged 18 years or older, based on the data from CHNS 1989–2011. The results showed striking decreases in the incidence and prevalence of hypertension based on the two-visit strategy for both males and females. Remarkably, the incidence of hypertension could be overestimated much more than prevalence when the one-visit strategy was adopted. In addition, when the two-visit strategy was adopted, the number of hypertension cases would decrease to 127.5–182.3 million or 254.0–374.1 million, according to the JNC 7 guideline criteria and the 2017 ACC/AHA guideline criteria, respectively.

The present data showed a decrease in the prevalence of hypertension by 47% among Chinese adults, based on CHNS 2006–2009/2011, which was equivalent to 127.5–182.3 million individuals. A similar trend was observed in other studies.^9^^,^^10^ The prevalence of hypertension based on the two-visit strategy was 35% lower than that based on the one-visit strategy in the Modesti et al study.^10^ In the Figueiredo^9^ et al study, the prevalence of hypertension based on the two-visit strategy was 11.2% lower than that based on the one-visit strategy. All of these showed that the prevalence would be overestimated when the one-visit strategy was used in the hypertension survey.

Because of differences in sampling, study design, and the definition of the two-visit strategy, the discrepancy between the one-visit strategy and the two-visit strategy varied widely in these studies. The decrease in our study was more notable than that in other studies. The time interval between the first visit and second visit might be an influential factor. In the Modesti and Figueiredo studies,^9^^,^^10^ the time interval between the first visit and the second visit was less than 35 days, whereas in our study, the second visit was conducted at least 2 years later. So the two-visit strategy in our study could eliminate the effect of high blood pressure (BP) variability, regression to the mean, and episodic hypertension better than the other studies could. Because of financial limitations or other reasons, implementation of the two-visit strategy in the hypertension survey was very difficult in large epidemiological studies, but continuous surveys for many years have been done in many countries, so our method was more practical to the actual situation. Owing to the long time interval between the first visit and the second visit, loss to follow-up in the two-visit strategy was inevitable, and sensitivity analyses were conducted in our studies. When the lost persons were treated as hypertensive patients, the prevalence of hypertension was still reduced by 25.5%.

Some studies have reported the impact of number of visits for blood pressure based on the prevalence of hypertension. In contrast, the incidence of hypertension in large populations was relatively scarce. In our study, a cohort study was conducted. Here, we showed that the incidence of hypertension decreased more than the prevalence based on the two-visit strategy, implying that the two-visit strategy could be used to identify hypertensive patients more accurately, especially in incident patients.

Aging and BMI were two contributors to the development of hypertension,^16^^–^^21^ so it was speculated that these two factors might have an effect on the discrepancy in the incidence (or prevalence) of hypertension between the one-visit strategy and the two-visit strategy. At the same time, the role of gender, smoking status, drinking status, region, and blood pressure levels were also analyzed. The results of the Cox’s models showed that old age, overweight/obesity, and higher blood pressure level were all significantly associated with an increased discrepancy in the incidence of hypertension between the one-visit strategy and two-visit strategy, especially among males, whereas the smoking status, drinking status, and region had no effect on the discrepancy. Similar results were observed in the population of Portugal. There are other important factors for hypertension, such as diabetes and salt intake, but the data on diabetes and salt intake could not been analyzed in this study because of lack of data.

The shift in the definition of hypertension from 140/90 mm Hg to 130/80 mm Hg for systolic/diastolic blood pressure is a major change in the 2017 ACC/AHA hypertension guidelines.^22^ It was indeed a shocking change, because the definition of hypertension was changed from 160/90 mm Hg to 140/90 mm Hg in the guideline of the JNC 5 published in 1993.^4^^,^^5^ Some studies focused on the Global Impact of 2017 ACC/AHA Guidelines in China and other populations,^23^^–^^27^ but reports of the effects of the two-visit strategy based on this new guideline were relatively scarce. So in our study, the JNC 7 and 2017 ACC/AHA high blood pressure guideline criteria were both adopted to predict the prevalence of hypertension based on the two-visit strategy in China. According to the JNC 7 high blood pressure guideline criteria, the number of hypertensives would decrease to 127.5–182.3 million when the two-visit strategy was adopted. Based on the 2017 ACC/AHA guideline, the corresponding number would decrease to 254.0–374.1 million. All these results showed that the burden of hypertension in China would decrease when the two-visit strategy was adopted in the hypertension survey.

### Limitations

First, approximately 20% of the hypertensive patients in the first visit were lost in the second visit in the cross-sectional study, which remains a source of potential follow-up bias. Thus, in our study, a sensitivity analysis was conducted to control for this bias. In addition, some social and environmental variables, such as physical activity, diet, and geographic regions might have had an impact on hypertension during the follow-up, while these factors were not considered in our study. These factors and their impacts on the discrepancy in the incidence and prevalence of hypertension between the one-visit strategy and the two-visit strategy should be analyzed in a future study. In this study, the results may be affected by the long interval between two blood pressure measurements of the two-visit strategy, while applying the two-visit strategy in our study could eliminate the effect of regression to the mean and episodic hypertension.

### Conclusions and relevance

The burden of hypertension would decrease from 244.5 million persons to 127.5–182.3 million persons in China if the two-visit strategy was applied, indicating that the health care burden of hypertension in 62.2–117.0 million patients could be averted.

## APPENDIX A. SUPPLEMENTARY DATA

eTable 1. Baseline characteristics of the participants who were free of hypertension: CHNS 1989–2006

eTable 2. Characteristics of the Participants Aged 18 Years and Older: CHNS 2006

eTable 3. Sensitivity Analysis of the hypertension prevalence^a^ based on two-visit strategy in 2006

eFigure 1. Flow chart illustrating the sample selection for the cohort study

eFigure 2. Flow chart illustrating the sample selection for the cross-sectional study
