# Supplementary material for: The Impact of Hypertension Definition Based on Two-visit Strategy on Estimate of Hypertension Burden: Results From the China Health and Nutrition Survey 1989–2011
Source: J Epidemiol. 2021 Mar 5;31(3):180–6. doi: 10.2188/jea.JE20190163 (PMC7878710; doi:10.2188/jea.JE20190163)
Supplement: Supplementary file 1 [file je-31-180-s001.pdf]

eTable 1. Baseline characteristics of the participants who were free of hypertension: CHNS 1989–2006

Characteristics	CHNS 1989-2009							Total
	1989	1991	1993	1997	2000	2004	2006	
All participants (n)	4764	3541	618	2180	1260	1136	558	14057
Age (years) [a]	31.7 (12.2)	48.0 (34.3)	26.6 (25.7)	33.7 (18.6)	33.8 (19.0)	37.9 (20.6)	35.5 (20.0)	34.0 (19.8)
BMI (kg/m^2) [a]	21.2 (3.0)	20.9 (3.5)	20.8 (2.9)	21.7 (3.7)	22.1 (3.9)	22.4 (4.1)	22.3 (4.5)	21.3 (3.5)
SBP (mmHg) [a]	110.0 (20.0)	110.0 (18.0)	110.0 (18.7)	113.3 (13.3)	113.3 (13.3)	115.3 (12.7)	114.8 (13.4)	110.0 (16.7)
DBP (mmHg) [a]	70.0 (12.0)	71.3 (12.7)	71.7 (12.7)	75.0 (10.0)	75.0 (10.0)	76.0 (10.0)	76.0 (10.0)	72.0 (12.0)
Ever smoking(%) [b]	26.3 (25.1-27.6) [c]	33.7 (32.2-35.3)	32.2 (28.5-35.9)	29.8 (27.9-31.7)	25.2 (22.8-27.6)	24.9 (22.4-27.4)	21.9 (18.4-25.3)	28.6 (27.9-29.3)
Ever drinking(%) [b]	30.7 (29.4-32.0) [c]	35.6 (34.0-37.2)	35.6 (31.8-39.4)	35.3 (33.3-37.3)	30.4 (27.9-32.9)	29.0 (26.4-31.7)	26.0 (22.3-29.6)	32.5 (31.7-33.3)
Urban resident(%) [b]	31.5 (30.2-32.8)	31.3 (29.8-32.9)	26.2 (22.7-29.7)	47.6 (45.5-49.7))	37.1 (34.4-39.7)	46.4 (43.5-49.3)	40.5 (36.4-44.6)	35.8 (35.0-36.6)
Male (n)	2220	1690	303	1005	505	464	208	6395
Age (years) [a]	31.8 (12.6)	47.0 (34.1)	29.0 (25.4)	34.1 (18.5)	36.7 (18.6)	40.1 (19.2)	38.7 (20.7)	34.8 (20.1)
BMI (kg/m^2) [a]	20.9 (2.8)	20.8 (3.1)	20.8 (2.8)	21.7 (3.6)	22.5 (4.2)	22.9 (4.1)	23.3 (4.6)	21.2 (3.4)
SBP (mmHg) [a]	110.0 (15.0)	112.0 (15.0)	110.7 (15.0)	118.0 (10.7)	118.7 (12.0)	120.0 (13.1)	119.3 (11.8)	113.7 (13.3)
DBP (mmHg) [a]	70.0 (12.0)	73.3 (10.7)	75.0 (10.0)	78.0 (10.0)	78.3 (10.0)	78.7 (10.0)	78.8 (8.7)	75.0 (10.0)
Ever smoking (%)[b]	54.9 (52.8-56.9) [c]	63.8 (61.6-66.1)	61.7 (56.2-67.2)	57.5 (54.5-60.6)	57.8 (53.5-62.1)	57.3 (52.8-61.8)	53.4 (46.6-60.1)	58.3 (57.1-59.6)
Ever drinking (%)[b]	53.7 (51.6-55.8) [c]	60.5 (58.1-62.8)	62.0 (56.6-67.5)	62.6 (59.6-65.6)	61.4 (57.1-65.6)	58.2 (53.7-62.7)	57.7 (51.0-64.4)	58.3 (57.1-59.6)
Urban resident(%) [b]	30.7 (28.8-32.6)	30.5 (28.3-32.7)	27.1 (22.1-32.1)	47.7 (44.6-50.7)	39.6 (35.3-43.9)	49.4 (44.8-53.9)	47.6 (40.8-54.4)	35.8 (34.6-36.9)
Female (n)	2544	1851	315	1175	755	672	350	7662
Age (years) [a]	31.6 (11.9)	48.4 (34.4)	25.2 (27.1)	33.1 (18.7)	31.8 (18.5)	35.7 (20.0)	33.6 (17.9)	33.4 (19.4)
BMI (kg/m^2) [a]	21.4 (3.2)	21.0 (4.0)	20.8 (3.3)	21.7 (3.9)	21.9 (3.8)	22.0 (4.0)	21.8 (4.3)	21.5 (3.7)
SBP (mmHg) [a]	109.0 (15.0)	110.0 (20.0)	108.0 (18.0)	110.0 (16.7)	110.0 (16.7)	110.7 (17.2)	110.7 (18.0)	110.0 (20.0)
DBP (mmHg) [a]	70.0 (12.0)	70.0 (13.7)	70.0 (12.7)	72.0 (10.7)	72.7 (11.3)	73.3 (11.3)	73.7 (12.0)	70.0 (13.3)
Ever smoking(%) [b]	1.5 (1.0-1.9) [c]	6.2 (5.1-7.3)	3.8 (1.7-5.9)	6.1 (4.8-7.5)	3.3 (2.0-4.6)	2.5 (1.3-3.7)	3.1 (1.3-5.0)	3.8 (3.4-4.2)
Ever drinking(%) [b]	10.7 (9.5-11.9) [c]	12.9 (11.3-14.4)	10.2 (6.8-13.5)	11.9 (10.1-13.8)	9.7 (7.6-11.8)	8.9 (6.8-11.1)	7.1 (4.4-9.8)	11.0 (10.3-11.7)
Urban resident(%) [b]	32.2 (30.4-34.0)	32.1 (30.0-34.2)	25.4 (20.6-30.2)	47.5 (44.6-50.3)	35.4 (32.0-38.8)	44.3 (40.6-48.1)	36.3 (31.2-41.3)	35.8 (34.7-36.9)

SBP, systolic blood pressure; DBP, diastolic blood pressure; BMI, body mass index.

[a] Median with Inter Quartile Range (IQR).

[b] Percentage with 95% confidence interval (95% CI).

[c] Because the percentages of ever smoker and alcohol drinker were not investigated in 1989, so we fill the missing value using the data from the same participants in 1991.

eTable 2. Characteristics of the Participants Aged 18 Years and Older: CHNS 2006

**Characteristics**	**Male**	**Female**
All participants (n)	4304	4823
Age (years) [a]	49.4 (21.6)	49.6 (21.4)
BMI (kg/m^2) [a]	22.8 (4.4)	22.8 (4.5)
SBP (mmHg) [a]	120.0 (19.4)	119.3 (21.3)
DBP (mmHg) [a]	80.0 (12.7)	78.0 (12.0)
Ever smoking (%)[b]	62.0 (60.6-63.5)	4.0 (3.4-4.6)
Ever drinking (%)[b]	58.4 (56.9-59.9)	8.2 (7.4-9.0)
Urban resident (%)[b]	34.2 (32.8-35.6)	34.2 (32.8-35.5)

BMI, body mass index; DBP, diastolic blood pressure; SBP, systolic blood pressure.

[a] Median with Inter Quartile Range (IQR).

[b] Percentage with 95% confidence interval (95% CI).

eTable 3. Sensitivity Analysis of the hypertension prevalence[a] based on two-visit strategy in 2006

Characteristics	Subtypes				HTN
	ISH	IDH	SDH	Med	
N	283	328	374	642	1627
All participants	2.25(1.94-2.55)	3.17(2.81-3.53)	3.08(2.73-3.44)	5.00(4.55-5.45)	13.50(12.80-14.20)
Gender					
Male	2.21(1.77-2.65)	4.26(3.66-4.87)	3.82(3.25-4.40)	4.68(4.05-5.31)	14.98(13.91-16.04)
Female	2.28(1.86-2.70)	2.18(1.77-2.60)	2.43(1.99-2.86)	5.32(4.69-5.95)	12.21(11.28-13.13)
Age (years)					
18-39	0.31(0.10-0.53)	2.00(1.46-2.54)	0.82(0.47-1.17)	0.31(0.10-0.53)	3.44(2.74-4.15)
40-59	1.95(1.54-2.37)	4.25(3.64-4.85)	4.17(3.57-4.77)	5.39(4.17-6.07)	15.76(14.67-16.85)
≥60	8.18(7.06-9.30)	4.00(3.20-4.80)	6.86(5.82-7.90)	16.99(15.45-18.53)	36.03(34.06-38.00)
BMI (kg/m^2)					
<18.5	1.16(0.28-2.05)	1.29(0.36-2.23)	2.59(1.28-3.90)	1.78(0.68-2.87)	6.83(4.74-8.91)
18.5-23.9	1.93(1.56-2.31)	2.59(2.15-3.02)	2.14(1.74-2.53)	3.14(2.66-3.61)	9.79(8.98-10.60)
24.0-27.9	2.22(1.65-2.79)	4.27(3.49-5.05)	3.36(2.66-4.05)	6.65(5.69-7.60)	16.49(15.06-17.92)
≥28.0	4.54(3.09-6.00)	5.55(3.95-7.14)	8.10(6.20-10.00)	11.31(9.10-13.52)	29.51(26.33-32.69)
Smoking status					
Never smoking	2.36(1.98-2.73)	2.81(2.40-3.22)	2.74(2.40-3.14)	5.29(4.74-5.84)	13.19(12.35-14.03)
Ever smoking	2.09(1.57-2.62)	3.96(3.24-4.67)	3.76(3.06-4.46)	4.46(3.71-5.22)	14.27(12.99-15.55)
Drinking status					
Never drinking	2.12(1.76-2.47)	2.57(2.17-2.96)	2.49(2.10-2.88)	5.32(4.76-5.88)	12.49(11.67-13.31)
Ever drinking	2.57(1.99-3.14)	4.39(3.64-5.13)	4.26(3.53-4.99)	4.13(3.41-4.85)	15.34(14.03-16.65)
Region					
Urban resident	1.93(1.44-2.41)	3.04(2.44-3.65)	2.80(2.22-3.38)	6.65(5.78-7.53)	14.42(13.19-15.66)
Rural resident	2.43(2.04-2.82)	3.24(2.79-3.69)	3.28(2.83-3.73)	4.05(3.55-4.55)	13.00(12.15-13.85)

BMI, body mass index, kg/m^2; CI, confidence interval; HTN, hypertension, total of the four subtypes; IDH, isolated diastolic hypertension; ISH, isolated systolic hypertension; Med, current use of antihypertensive medication; SDH, systolic-diastolic hypertension.

[a] the prevalence based on two-visit strategy = (the population of hypertension based on two- visit strategy + the lost population based on two-visit strategy)/all the population in 2006

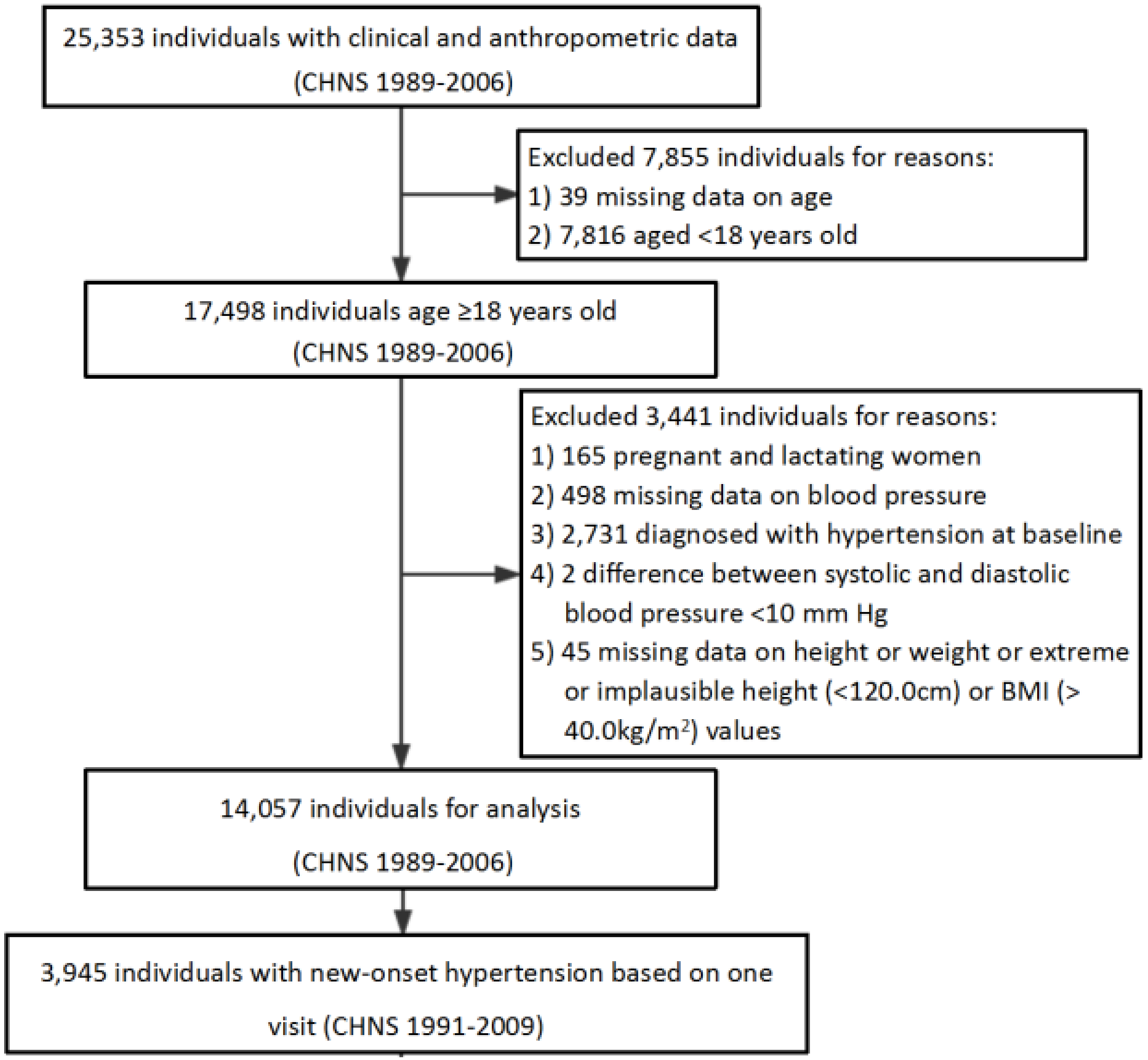

eFigure 1. Flow chart illustrating the sample selection for the cohort study

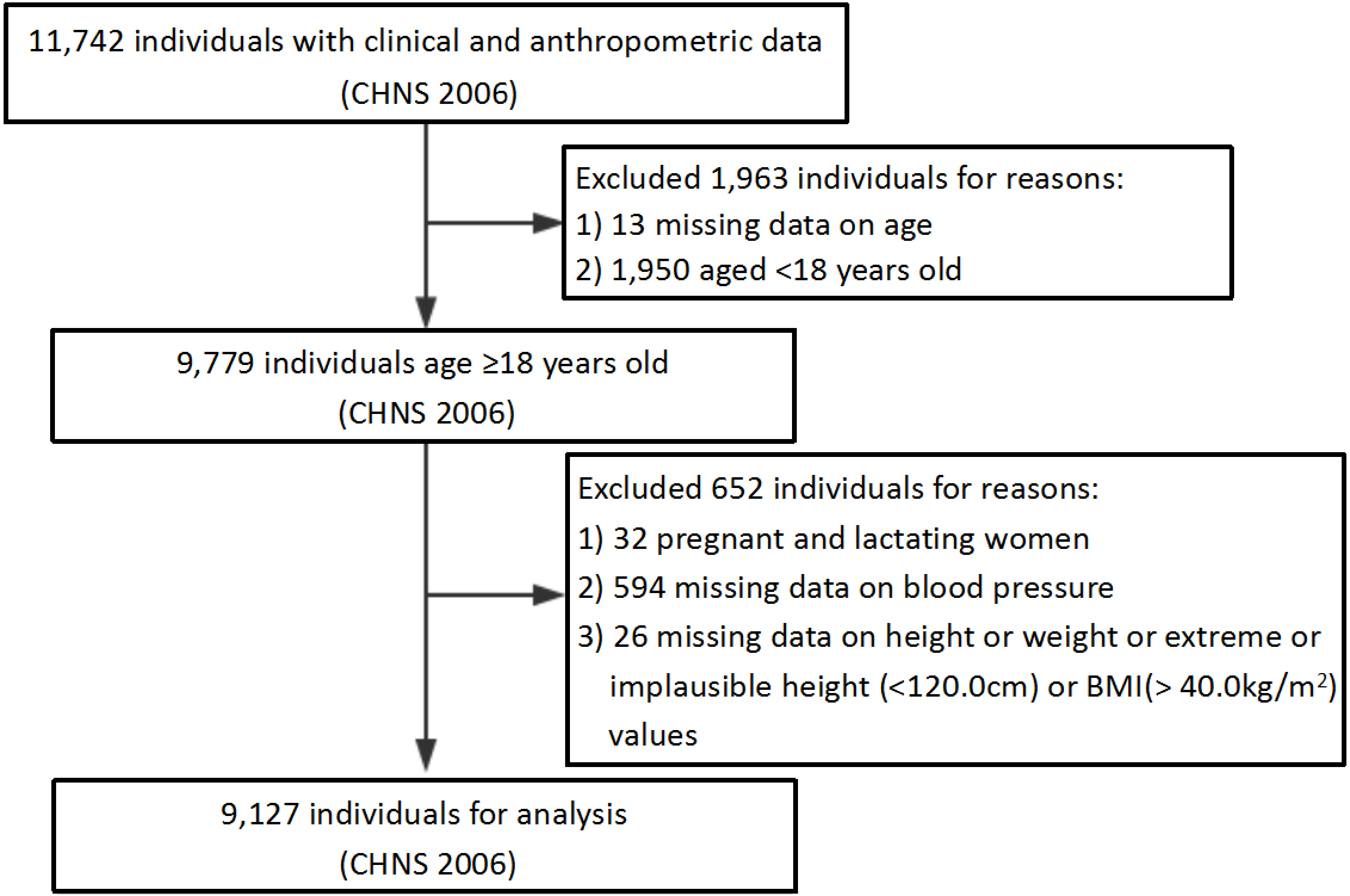

eFigure 2. Flow chart illustrating the sample selection for the cross-sectional study
